# Surgical management of a PICC-associated right atrial thrombus: a case report

**DOI:** 10.3389/fcvm.2026.1772698

**Published:** 2026-08-26

**Authors:** Tietuo Jin, Rui Wang, RuiJuan Zhang, Gaofeng Pan, Liang Dong

**Affiliations:** 1 Department of Intensive Care Medicine, Taizhou Central Hospital (Taizhou University Hospital), Taizhou, Zhejiang, China; 2 Department of Dermatology, Taizhou Central Hospital (Taizhou University Hospital), Taizhou, Zhejiang, China; 3 Department of Cardiothoracic Surgery, Taizhou Central Hospital (Taizhou University Hospital), Taizhou, Zhejiang, China

**Keywords:** breast cancer, case report, PICC, right atrial thrombus, surgical thrombectomy

## Abstract

A patient who had undergone surgery for breast cancer received a peripherally inserted central catheter (PICC) for chemotherapy. Three months later, she developed chest tightness and dyspnoea. Transthoracic echocardiography and computed tomography revealed a right atrial mass, with the tip of the PICC positioned within the right atrium. After 1 month of anticoagulation with rivaroxaban, the right atrial lesion showed no appreciable reduction in size, and the patient ultimately underwent surgical treatment. Intraoperatively, the mass was found to be closely adherent to the right atrial wall and not connected to the PICC. Histopathological examination demonstrated a mixed thrombus, predominantly composed of fibrinoid material.

## Introduction

Peripherally inserted central catheters (PICCs) are widely used to deliver chemotherapy in patients with cancer (1, 2). Venous thrombosis is among the most common complications of PICC placement and may lead to life-threatening events (3). Reports of PICC-associated right atrial thrombus are limited; such thrombi carry a higher risk of pulmonary embolism, and there is no consensus regarding the optimal management strategy, particularly the timing of surgical intervention. In the present case, catheter tip malposition into the right atrium was followed by the development of a large mixed right atrial thrombus within 3 months. Standard anticoagulation with rivaroxaban was ineffective, and surgical thrombectomy was ultimately performed. Intraoperatively, the thrombus was found to be tightly adherent to the right atrial wall and not connected to the PICC; histopathology showed a fibrin-rich mixed thrombus, a feature that is rarely described in prior case reports. This case may provide practical insights into the diagnosis and management of PICC-associated right atrial thrombosis.

## Case presentation

**Figure 1:**
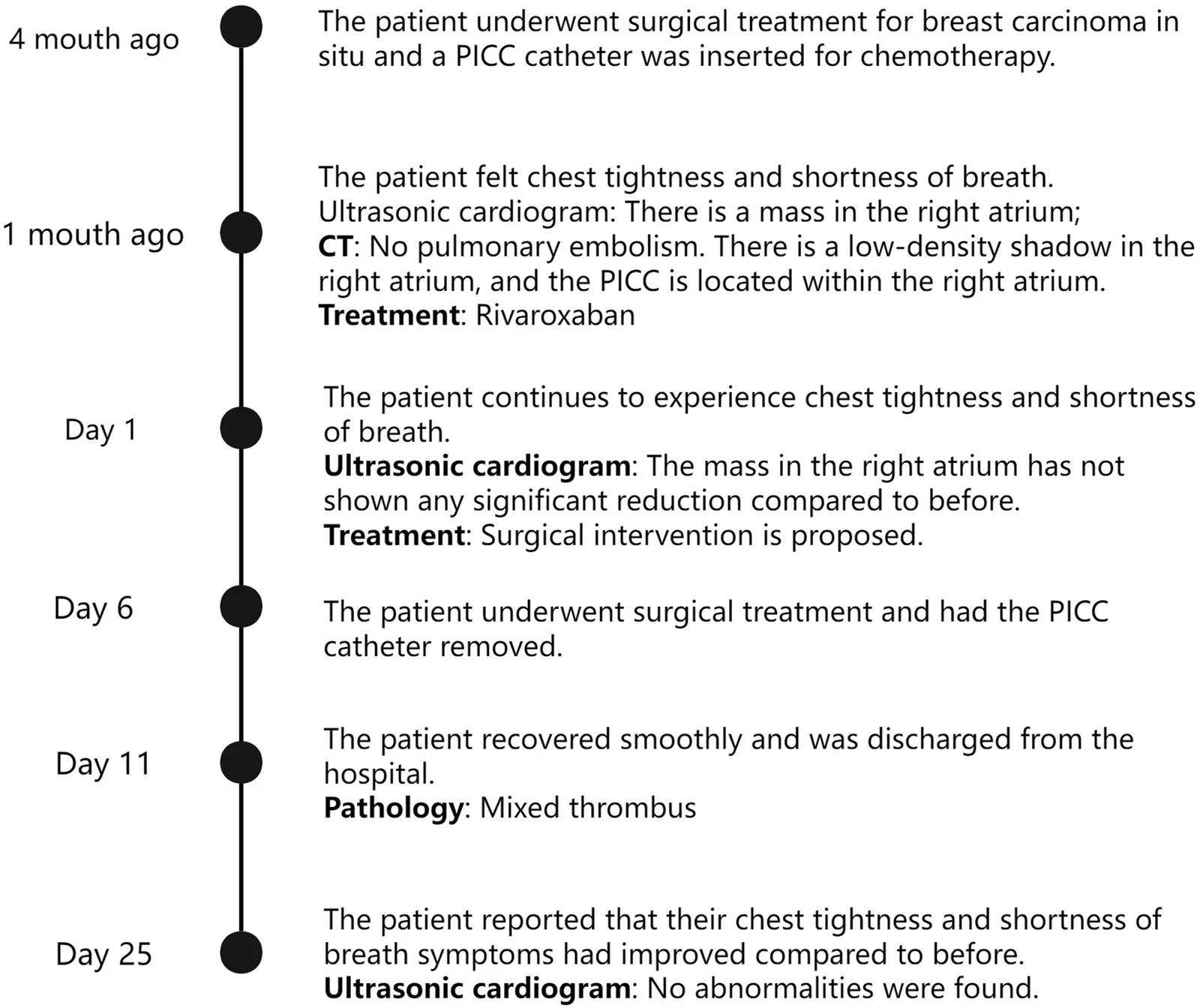
Multimodal imaging of a right atrial mass associated with PICC malposition. **(A,B)** Transthoracic echocardiography demonstrates a hyperechoic, relatively fixed right atrial mass measuring 22.83 × 25.95 mm, located approximately 9 mm from the tricuspid valve annulus. **(C,D)** Computed tomography confirms the PICC tip within the right atrium and shows a concomitant low-attenuation mass in the right atrium.

**Figure 2:**
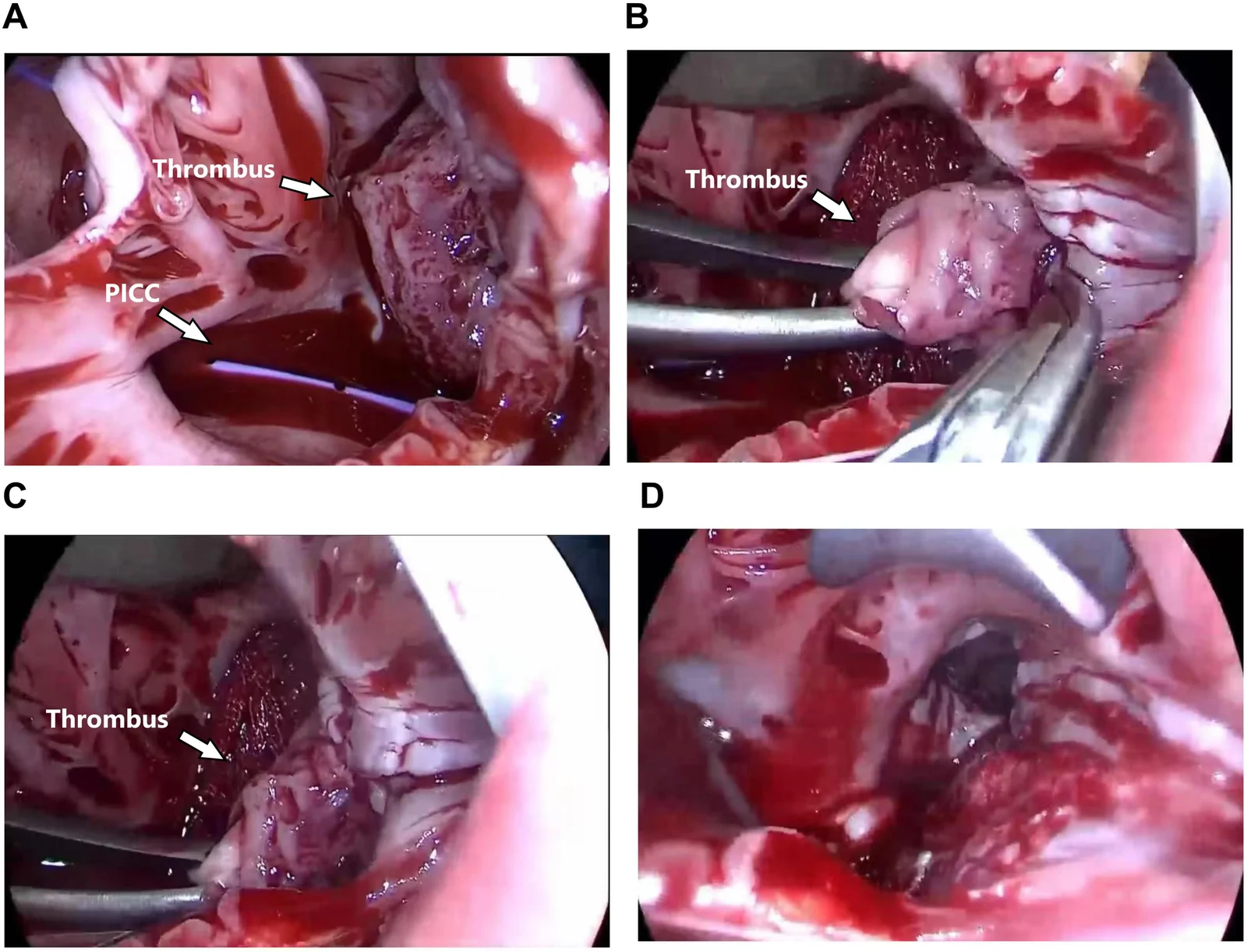
Intraoperative findings during surgical thrombectomy. **(A)** The PICC tip and the right atrial thrombus are visualised within the right atrium; the thrombus is not in continuity with the catheter. **(B,C)** The thrombus is firmly adherent to the right atrial wall. **(D)** The right atrium after complete thrombus removal.

**Figure 3:**
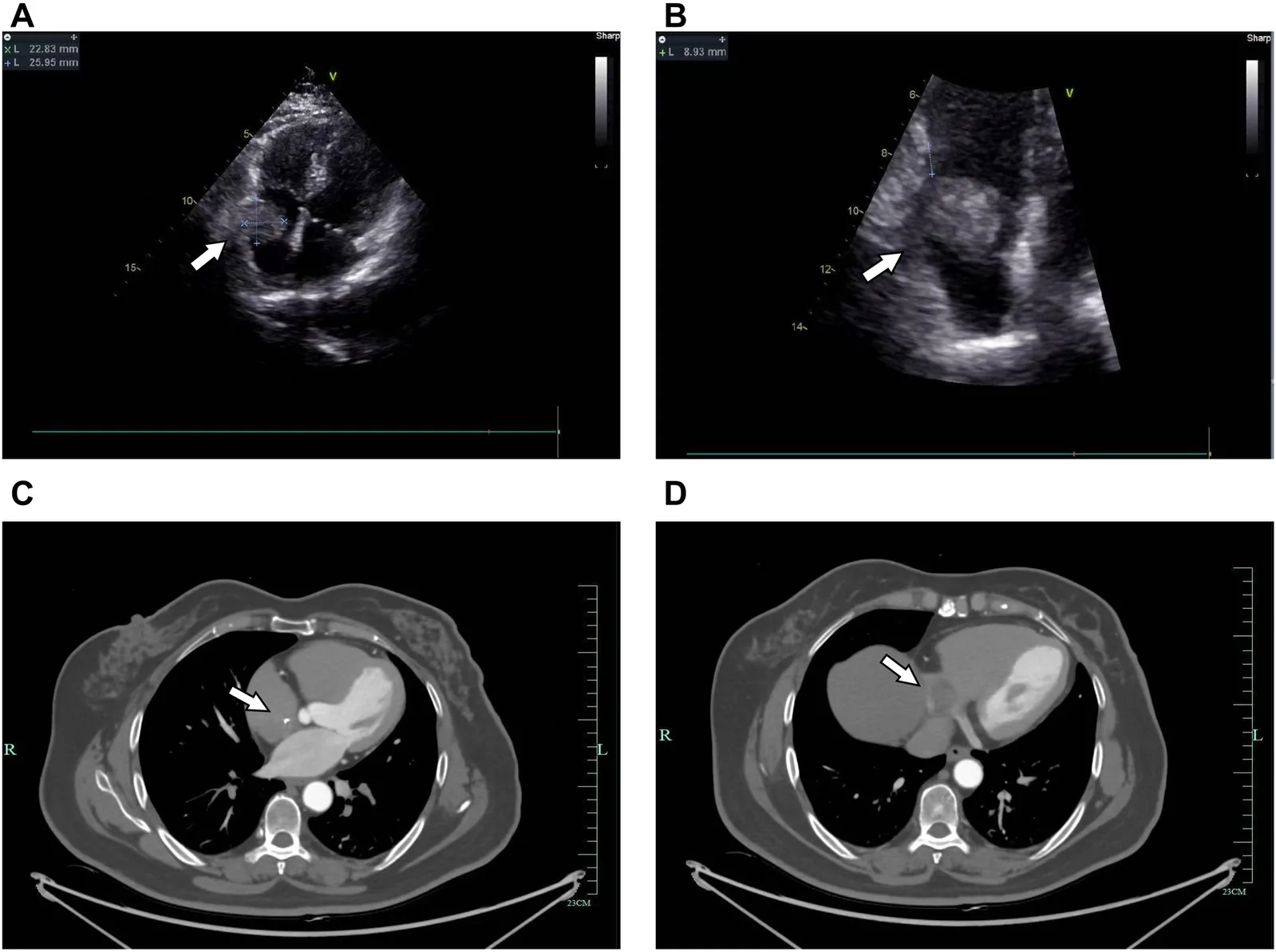
Histopathology and postoperative follow-up imaging. **(A)** Histological examination of the excised mass demonstrates a mixed thrombus predominantly composed of fibrinous (fibrinoid) material, with only scant red blood cells and inflammatory cells. **(B)** Transthoracic echocardiography at postoperative day 20 shows no residual right atrial mass.

**Figure 4:**
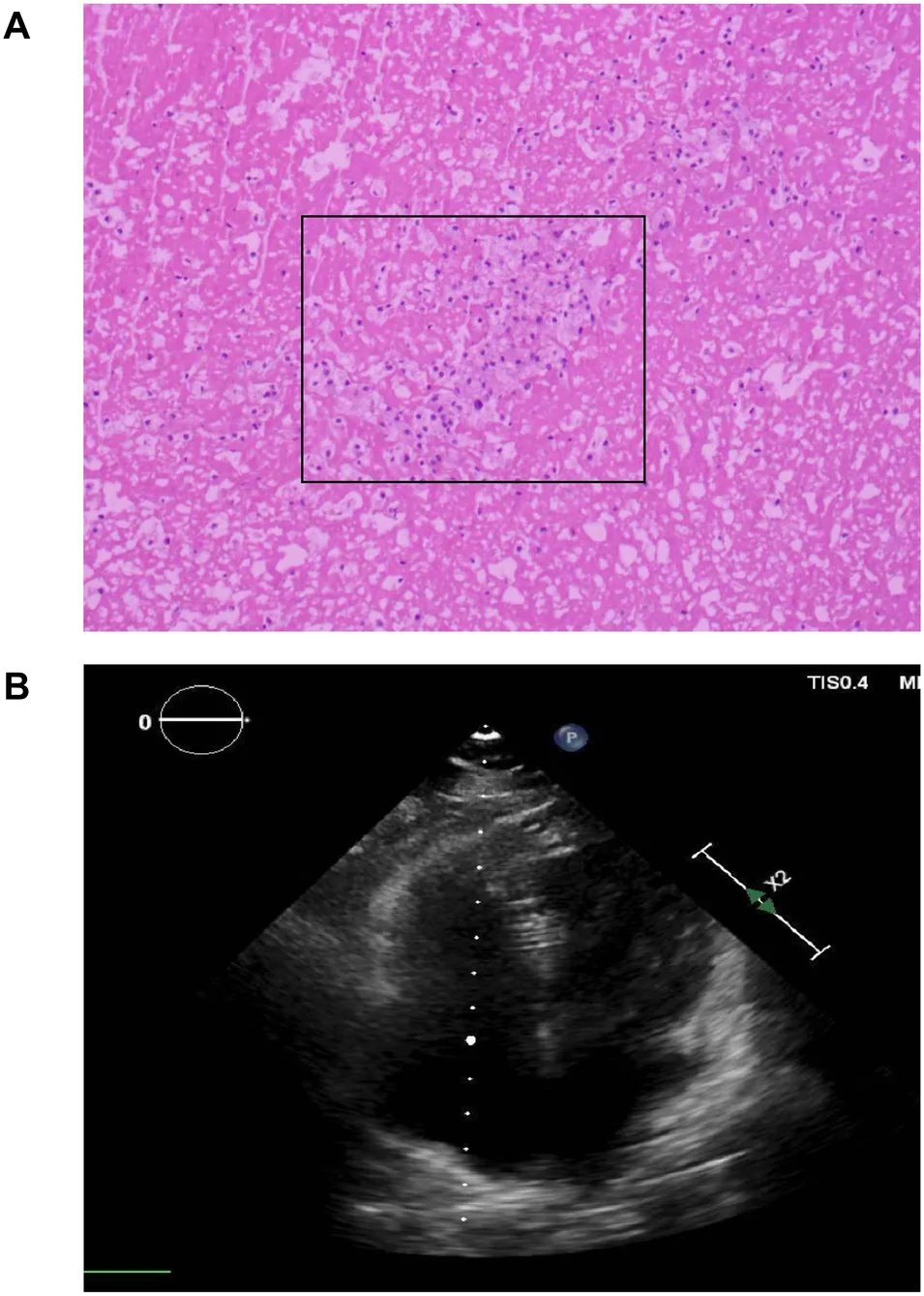
Timeline of the patient's clinical course.

A 51-year-old woman has a 2-year history of type 2 diabetes mellitus, treated long-term with metformin and with good glycaemic control. Four months earlier, she had undergone surgery for ductal carcinoma *in situ* (DCIS) of the breast; preoperative transthoracic echocardiography showed no significant abnormalities. A peripherally inserted central catheter (PICC) was placed postoperatively to facilitate adjuvant chemotherapy with four cycles of TC (docetaxel + cyclophosphamide). We were unable to obtain imaging data regarding the position of the PICC tip. After discharge, the patient underwent weekly PICC maintenance at the PICC clinic, during which heparin was used. She had no family history of diabetes or malignancy. One month before presentation, she developed chest tightness and dyspnoea, which worsened with exertion and improved with rest, and was exacerbated in the left lateral decubitus position. Her symptoms were consistent with New York Heart Association (NYHA) functional class II. On examination, cardiac auscultation revealed no audible murmurs or extra heart sounds. Breath sounds were clear bilaterally, without rales. Transthoracic echocardiography demonstrated a relatively fixed right atrial mass measuring approximately 22.83 × 25.95 mm, with mild tricuspid regurgitation and a left ventricular ejection fraction (LVEF) of 58%. Transesophageal echocardiography did not reveal a patent foramen ovale. Coronary computed tomography angiography (CTA) showed a low-attenuation lesion in the right atrium and confirmed that the tip of the PICC was located within the right atrium (Figure 1). Pulmonary CTA showed no evidence of pulmonary embolism. The patient was afebrile, and laboratory testing did not support an active infection (White blood cell count (WBC): 5.5 × 10⁹/L, Absolute neutrophil count (ANC): 3.7 × 10⁹/L, High-sensitivity C-reactive protein (hs-CRP): 3.4 mg/L, Procalcitonin (PCT): 0.043 ng/mL). The main diagnostic challenge was the uncertain nature of the right atrial mass. Although right atrial metastasis from breast cancer has been reported, her underlying malignancy was ductal carcinoma *in situ*, making cardiac metastatic disease unlikely (4). We therefore favoured a diagnosis of PICC-associated thrombus, and initiated oral rivaroxaban anticoagulation (days 1–21, 15 mg twice daily; from day 22 onward, 20 mg once daily). After 1 month of treatment, her symptoms showed no meaningful improvement, and follow-up echocardiography demonstrated no appreciable change in the size of the right atrial lesion. To further clarify the diagnosis and mitigate the potential risk of embolic events, we proceeded with surgical management. The reason we decided to perform open heart surgery instead of transcatheter thrombus aspiration for the patient was that the nature of the mass could not be definitively determined as thrombus preoperatively. During initiation of cardiopulmonary bypass, the activated clotting time (ACT) persistently failed to reach the target of 480 s despite administration of more than 40,000 IU of heparin; the ACT increased only after transfusion of fresh frozen plasma. These findings were suggestive of heparin resistance, although antithrombin levels were not measured. Intraoperatively, a friable mass was identified on the lateral wall of the right atrium. The lesion was firmly adherent to the atrial wall and measured 30 × 20 × 5 mm. The tip of the PICC was located adjacent to the mass but was not connected to it (Figure 2). The mass was excised and the PICC was removed. Frozen-section examination revealed fibrinoid necrotic material, and the final pathology confirmed a mixed thrombus composed predominantly of fibrinoid tissue, with only scant erythrocytes and inflammatory cells (Figure 3A). Accordingly, the diagnosis was established as right atrial thrombosis. The postoperative course was uneventful, and the patient was discharged on oral rivaroxaban 10 mg once daily for 14 days. At 20 days after surgery, she reported substantial improvement in chest tightness, with NYHA functional class I symptoms, and follow-up transthoracic echocardiography showed no abnormalities (Figure 3B). Figure 4 summarises the patient's treatment course in a timeline.

## Discussion

According to the literature, the incidence of PICC-associated deep vein thrombosis (DVT) is approximately 2%–5%, whereas right atrial involvement has been reported far less frequently (5, 6). Proposed risk factors include an abnormal catheter tip position (for example, within the right atrium), prolonged catheter dwell time, underlying conditions such as malignancy and hypercoagulability, and comorbidities including diabetes mellitus and hypertension (7).

Although previous reports have described PICC-associated right atrial thrombosis, this case presents several unique features that expand the existing literature. First, to the best of our knowledge, this is one of the few cases providing a detailed histopathological characterization of such a thrombus, allowing us to observe resistance to rivaroxaban therapy in a mixed right atrial thrombus composed predominantly of fibrin with minimal red blood cell content. Second, we describe intraoperative heparin resistance observed for the first time in this specific clinical context, which was temporarily reversed by fresh frozen plasma transfusion. Third, intraoperative findings revealed that the thrombus was firmly adherent to the atrial wall without attachment to the catheter tip, challenging the common assumption that catheter-directed thrombus is necessarily connected to the catheter and suggesting the possibility of local thrombus formation induced by mechanical stimulation alone.

Beyond the classic Virchow's triad, the unique biological context of an indwelling catheter introduces additional mechanistic complexity. The catheter itself serves as a foreign surface that rapidly adsorbs plasma proteins—particularly fibrinogen and contact factors—triggering the contact activation pathway (factors XII and XI), amplifying platelet adhesion and activation, and initiating the coagulation cascade (8). Simultaneously, catheter insertion causes direct mechanical injury to the endothelium—whether at the venipuncture site or, as in our patient, at the catheter tip due to malposition against the right atrial wall—exposing subendothelial tissue factor and further propagating thrombin generation. The low-pressure, relatively low-flow environment of the right atrium may promote local blood stasis, reducing the dilution and washout of activated coagulation factors and further facilitating thrombus growth. The PICC and the evolving thrombus may also disturb local haemodynamics, generating vortical flow that further promotes thrombogenesis. In addition, cancer-associated hypercoagulability can accelerate catheter-related thrombosis, a phenomenon not uncommon in breast cancer; prior data suggest a 7.0% incidence of PICC-related thrombosis in breast cancer patients, with elevated fibrinogen, increased platelet counts and catheter malposition identified as key risk factors (9). Over time, the initially soft thrombus matures into an organized, fibrin-rich structure through fibroblast proliferation and extracellular matrix remodeling, while local fibrinolysis becomes impaired—the dense fibrin-collagen matrix impedes penetration of plasminogen activators, and concurrent upregulation of plasminogen activator inhibitor-1 further suppresses fibrin degradation (10). This dynamic evolution from acute platelet-fibrin deposition to organized fibrin-rich thrombus explains why a lesion that forms rapidly may later become resistant to pharmacological dissolution, as the structural and functional properties of the thrombus change fundamentally over time. Finally, the apparent heparin resistance improved after transfusion of fresh frozen plasma, strongly suggesting possible antithrombin deficiency, although antithrombin activity was not measured; antithrombin deficiency is an important risk factor for thrombosis (11). We acknowledge the lack of antithrombin level measurement as a significant limitation; therefore, this observation should be considered hypothesis-generating rather than confirmatory. Future studies of PICC-associated thrombosis may benefit from routine assessment of antithrombin activity, particularly in cases demonstrating heparin resistance. The TC chemotherapy regimen may be associated with thrombus formation (12). The lack of FDG-PET/CT to determine whether the right atrial mass was a metastatic lesion is a limitation. Regarding long-term prognosis, the patient's DCIS carries an excellent survival rate, and right atrial thrombectomy has a minor lasting impact on cardiac function.

We could not determine whether this case represented true anticoagulation failure because the nature of the right atrial lesion could not be definitively established preoperatively. Surgical intervention was pursued for three reasons: the mass was large and highly suspicious for thrombus, conferring a substantial risk of pulmonary embolism; the response to anticoagulation was unsatisfactory; and the patient developed symptoms of cardiac dysfunction, raising concern that the right atrial mass was compromising haemodynamics. Postoperative histopathology confirmed a mixed thrombus; however, it was predominantly composed of fibrinoid/organised tissue with only scant erythrocytes and inflammatory cells. Rivaroxaban exerts its therapeutic effect by directly inhibiting coagulation factor Xa, reducing thrombin generation, thereby blocking fibrin formation, inhibiting new thrombus growth, and preventing expansion of the existing thrombus. It primarily targets ongoing coagulation; however, the organized, fibrin-rich thrombus found intraoperatively was structurally independent of active coagulation, explaining why one month of rivaroxaban therapy failed to reduce it (13). An alternative explanation for the lack of response to rivaroxaban is that the duration of anticoagulation (1 month) may have been insufficient, particularly for an organized thrombus. Some reports suggest that 6–8 weeks of anticoagulation may be required to observe regression of catheter-related thrombus (14). However, given the densely organized, fibrin-rich histology in our patient, we argue that even prolonged therapy would likely have been ineffective.

In addition to mechanistic considerations, our management strategy should be placed within the broader context of individualized antithrombotic therapy. While anticoagulation is the standard first-line approach for catheter-related thrombosis, clinical decision-making must carefully weigh the competing risks of thromboembolic complications and bleeding. Recent evidence on predictors of major bleeding during anticoagulant therapy highlights the importance of incorporating patient-specific factors into therapeutic decisions (15). Our case exemplifies this principle: the decision to proceed with surgical thrombectomy rather than prolonged anticoagulation or catheter-directed thrombolysis was reached after multidisciplinary evaluation, taking into account the high thrombotic burden, the organized nature of the lesion, the limited likelihood of response to further medical therapy, and the patient's overall surgical fitness. We advocate that similar cases should be managed with a patient-centered approach, integrating both thrombotic and bleeding risk stratification to guide optimal therapy.

## Limitations

Several limitations should be acknowledged. First, the follow-up period was relatively short (20 days post-discharge), and long-term echocardiographic surveillance for thrombus recurrence was not available, although the patient remained asymptomatic at the last follow-up. Second, antithrombin activity was not measured; therefore, the observed heparin resistance remains hypothesis-generating. Third, FDG-PET/CT was not performed to definitively exclude right atrial metastasis, though the clinical context made this unlikely. Fourth, the postoperative anticoagulation duration was only two weeks, which deviates from the recommended minimum of three months after surgical thrombectomy—this is a major limitation, and this abbreviated regimen should not be followed. Future studies should include routine antithrombin monitoring and longer follow-up to better define optimal management.

## Conclusion

This case underscores the importance of vigilant PICC management in patients with cancer, including regular verification of catheter tip position and prompt correction of malposition to reduce thrombotic risk. When an intracardiac mass is detected in a patient with a PICC, catheter-associated right atrial thrombus should be considered a key differential diagnosis. While no consensus exists on optimal management, surgical thrombectomy may be considered when anticoagulation proves ineffective or symptoms persist. Postoperative anticoagulation should follow recommended guidelines, and the abbreviated regimen used in this case is not advised.

## Data Availability

The original contributions presented in the study are included in the article/Supplementary Material, further inquiries can be directed to the corresponding author.
